# Coverage of Native Plants Is Key Factor Influencing the Invasibility of Freshwater Ecosystems by Exotic Plants in China

**DOI:** 10.3389/fpls.2018.00250

**Published:** 2018-02-28

**Authors:** Haihao Yu, Ligong Wang, Chunhua Liu, Shufeng Fan

**Affiliations:** The National Field Station of Freshwater Ecosystem of Liangzi Lake, College of Life Science, Wuhan University, Wuhan, China

**Keywords:** biological invasion, biodiversity, aquatic vegetation, eutrophication, global change

## Abstract

Understanding the biotic and abiotic factors that influence the susceptibility of a community to invasion is beneficial for the prediction and management of invasive species and the conservation of native biodiversity. However, the relationships between factors and invasibility of a community have not been fully confirmed, and the factors most associated with the susceptibility of a community to invasion have rarely been identified. In this study, we investigated the species richness patterns in aquatic exotic and native plants and the relationships of exotic species richness with habitat and water environment factors in 262 aquatic plant communities in China. A total of 11 exotic plant species were recorded in our field survey, and we found neither a negative nor a positive relationship between aquatic exotic and native plant species richness. The aquatic exotic plant species richness is negatively correlated with the relative coverage and biomass of native plants but positively correlated with the total nitrogen (TN), total phosphorus (TP), and chemical oxygen demand (COD) concentrations in the water. The native plant species richness, native species’ relative coverage, and native species’ biomass were positively related to each other, whereas the TP, TN, and COD were also positively related to each other. The native plant species richness, native species’ relative coverage, and native species biomass were each negatively correlated with the TP, TN, and COD. In addition, biotic rather than abiotic predictors accounted for most of the variation in exotic plant richness. Our results suggest that improving the vegetation coverage and the biodiversity of native plants is the most effective approach for preventing alien plant invasions and minimizing their impacts on freshwater ecosystems.

## Introduction

Plant invasions reduce biodiversity and alter the species composition, structures, processes, and functions of invaded ecosystems (Vitousek, 1990; D’Antonio and Kark, 2002; Vilà et al., 2011). Moreover, invasions are considered one of the main causes of species extinctions (Clavero and García-Berthou, 2005). Understanding the mechanism of invasion can help control biological invasions and is also beneficial to biodiversity conservation. Some biotic and abiotic factors influence the susceptibility of a community to invasion; however, the relationships between these factors and the invasibility of a community have not been fully confirmed. In addition, identifying the factors most closely associated with the susceptibility of a community to invasion has rarely been investigated.

Communities with high biodiversity are considered more capable of resisting invasions (Elton, 1958; Kennedy et al., 2002) because more species produce stronger competition pressure and more fully occupy the available niche space, thereby limiting the opportunities for additional species to establish and survive (Elton, 1958; Levine and D’Antonio, 1999; Shea and Chesson, 2002). Previous studies have found the existence of negative correlations between native species richness and invasive richness at fine scales (Naeem et al., 2000; Kennedy et al., 2002; Gilbert and Lechowicz, 2005). In contrast, rich native areas support many more invasive species than areas with relatively few native species at the landscape or regional scales (Lonsdale, 1999; Fridley et al., 2007). Habitat heterogeneity or the spatial variability of resources or conditions at large scales promotes the coexistence of native and exotic species. Moreover, sites with favorable growing conditions generate high richness in both native and exotic species (Stohlgren et al., 2006; Fridley et al., 2007). Previous studies have also reported that native and exotic species richness values are positively related at both the local and the landscape scales (Davies et al., 2007; Souza et al., 2011), although no relationship between native and invasive species richness has been found (Capers et al., 2007).

Resource availability is a key factor that determines the susceptibility of a community to invasion by exotic species. The fluctuating resources hypothesis states that a plant community becomes more susceptible to invasion whenever there is an increase in the amount of unused resources (Davis et al., 2000). Invasive plants can outperform native plants in resource-rich environments (Daehler, 2003; Burns, 2004; Blumenthal, 2005; Fan et al., 2013). However, invasive plants can exhibit greater performance, resource-use efficiency, and phenotypic plasticity than native plants in resource-limited environments (Funk and Vitousek, 2007; Funk, 2008; Matzek, 2011). Other elements of global change can also affect the success of biological invasion (Dukes and Mooney, 1999), but whether these elements facilitate or inhibit the invasion of plants still needs to be clarified. For instance, increased atmospheric CO_2_ concentrations can positively or negatively impact invasive plants (Dukes and Mooney, 1999), and warmer climate can also have diametrically opposite effects on the potential ranges of invasive plants (Merow et al., 2017).

Freshwater ecosystems are particularly vulnerable to invasion (Shea and Chesson, 2002). Greater rates of commerce lead to high propagule pressure in freshwater ecosystems, and propagules also spread rapidly in connected water systems. Furthermore, freshwater ecosystems are one of the most severely altered ecosystems due to human activity. During the past 30 years in China, rapid urbanization, gross domestic product (GDP) increases, vast population growth, and living standard improvements have all produced domestic and industrial wastewater. Moreover, due to insufficient sewage treatment capacities, some of this wastewater is discharged without treatment directly into rivers and lakes (Shao et al., 2006; Yang and Pang, 2006; Le et al., 2010), which causes high levels of organic compounds and nutrients (nitrogen and phosphorus) in rivers and lakes and thus the organic pollution and eutrophication of many water bodies. Recently, 73% of the major lakes in China have undergone severe eutrophication, and the area of eutrophication amounts to 15,000 km^2^ (Jin et al., 2005; Li, 2006). Land-use and land-cover changes, environmental pollution, overexploitation, and large hydroelectric projects have severely reduced lake sizes, increased habitat fragmentation, decreased both richness and coverage of aquatic vascular plants, altered the species composition of aquatic plant communities, and reduced the population size and individual body size of some species in the freshwater ecosystems of China (Fang et al., 2006). These changes may affect the invasion of exotic aquatic plants. In this study, we investigated the relationships among native plant richness, native vegetation coverage, native vegetation biomass, water nutrition [total nitrogen (TN) and total phosphorus (TP) concentrations], pollution level [chemical oxygen demand (COD)], habitat size, latitude, and exotic plant richness, and determined the correlations among these factors in the freshwater ecosystems of China. We also identified the explanatory power of these factors for the richness of exotic plant species in communities.

## Materials and Methods

### Field Surveys

**FIGURE 1:**
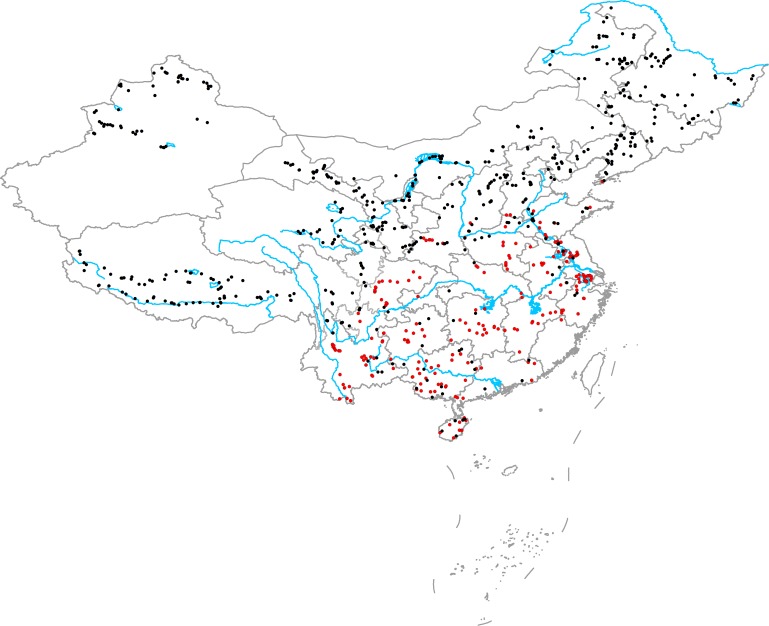
Geographical locations of the sampling sites (red circles show the communities in which at least one exotic plant was present and black circles show the communities that did not have exotic plants).

From June to October in 2014, 2015, and 2016, we surveyed 594 aquatic plant communities in various natural freshwater ecosystems (including brackish lakes) throughout China. The survey area included northeast China, northwest China, western China, eastern China, northern China, central China, southern China, southwest China, Hainan Island, and Qinghai Tibet Plateau (**Figure 1**). In high latitude and altitude regions (northeast China, northwest China, northern China, Qinghai Tibet Plateau), plant communities were surveyed from June to August. In the middle and low latitude and altitude regions (eastern China, northern China, central China, southern China, southwest China, Hainan Island), plant communities were surveyed from August to October. After selecting a community, we first recorded the habitat information: location (GPS), habitat type (lake, river, canal, marsh, pond, or reservoir), and habitat size (the width of river and canal or the shortest distance between the two banks). We then placed 9–20 quadrants (1 m × 1 m) along three-to-six transects, and more quadrants and transects were placed in large or complicated communities. In large, simple communities, the distances between two adjacent quadrants and transects were large (30, 50, 100, and 200 m), whereas the distances between two adjacent quadrants and transects in complicated communities were smaller (5, 10, and 20 m). Briefly, the quadrants in a community were selected to represent the actual community structure. For each quadrant, the relative coverage of the native plants (*C*) was determined by visual estimation, and all native plants (including emergent and submerged parts and roots) and vegetative propagules in the quadrants were collected, washed, and dried at 70°C for >48 h to determine the total biomass (*M*). The mean relative coverage of native plants in the community (*Cn*) was calculated as follows:

$$Cn=\frac{C1+C2+...+Ci}{i},$$

where *i* is the number of quadrants in the community.

The mean biomass of the native plants in the community (*Mn*) was calculated as follows:

$$Mn=\frac{M1+M2+...+Mi}{i},$$

where *i* is the number of quadrants in the community.

We recorded all exotic and native plants in the community, including species that were not present in the quadrants. A water sample was collected in the center of the community, and the TN and TP concentrations as well as the COD in the water were determined with a Palintest 7500 Photometer (Palintest, United Kingdom).

### Data Analyses

A total of 594 aquatic plant communities and 5583 quadrants were surveyed in various natural freshwater ecosystems throughout China. However, only 262 communities in the main distribution area of exotic plants were analyzed. We applied simple linear regressions to determine the patterns of exotic plant richness along environmental and biological gradients, including the richness, relative coverage and biomass of native plants, latitude, habitat size, and TN, TP, and COD contents of the water bodies. In addition, simple linear regressions were performed among these environmental and biological factors. To identify which biotic or abiotic factors are most associated with exotic plant richness, we employed hierarchical partitioning to analyze the independent explanatory power (*I*) of these factors for the richness of exotic plant species. The statistical significance of each *I* was determined by randomizing the data matrix 100 times (Mac Nally, 2002). The results of the significance tests are expressed as *Z*-scores. The linear regressions were performed using SPSS 13.0 (SPSS Inc., Chicago, IL, United States). The hierarchical partitioning was performed using the hier.part package (Walsh and Mac Nally, 2013) in R v3.4.2 (R Development Core Team, 2017).

## Results

There were 222 communities in which at least one exotic plant was present and 372 communities where no exotic plants were present. In the main distribution area of exotic plants (eastern China, central China, southern China, southwest China, and Hainan Island), only 40 communities did not have exotic plants. In the 262 communities in the main distribution area of exotic plants, a total of 11 exotic plant species were recorded: *Alternanthera philoxeroides*, *Eichhornia crassipes*, *Pistia stratiotes*, *Myriophyllum aquaticum*, *Hydrocotyle vulgaris*, *Egeria densa*, *Elodea nuttallii*, *Cabomba caroliniana*, *Echinodorus* sp., *Nuphar* sp., and *Azolla filiculoides*. The richness of exotic plant species in the 262 communities ranged from 0 to 4 species, and the richness of the native plant species in the 262 communities ranged from 1 to 27 species.

**FIGURE 2:**
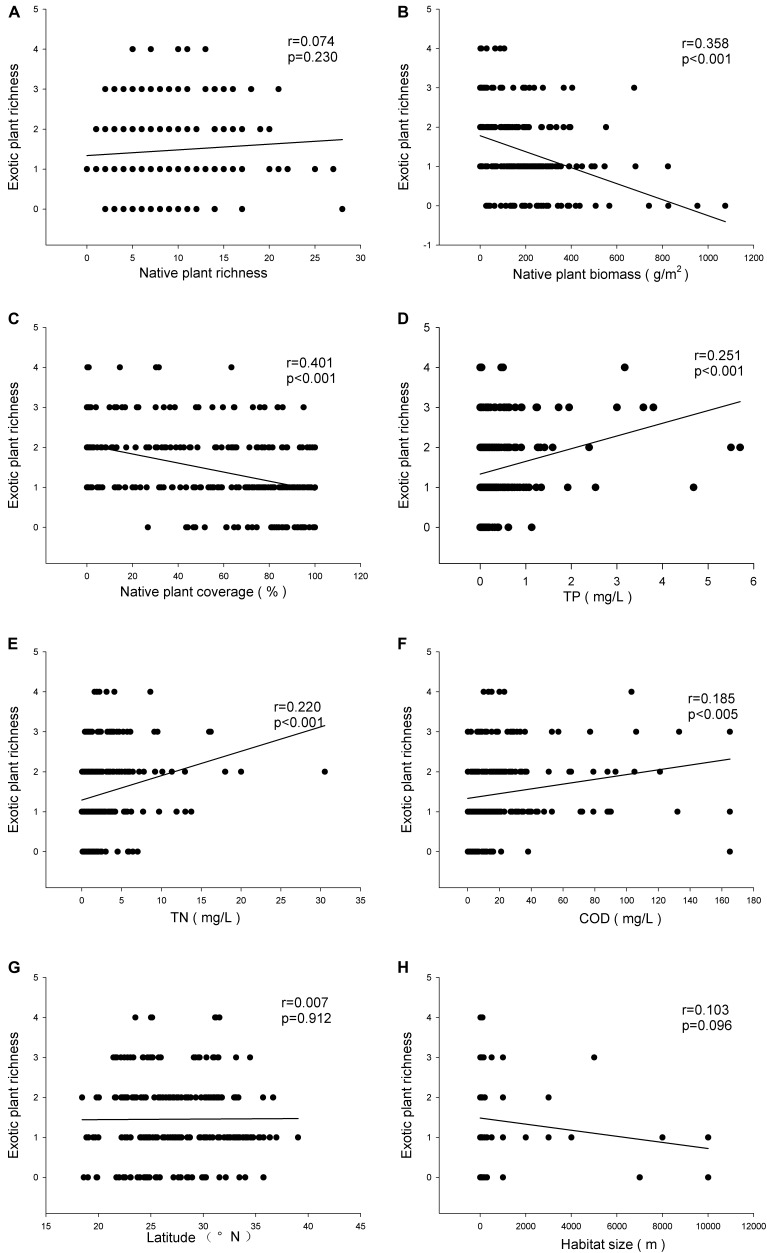
Relationships of invasive plant richness with native plant richness **(A)**, mean biomass of native plants **(B)**, relative coverage of native plants **(C)**, TP **(D)**, TN **(E)**, COD **(F)**, latitude **(G)**, and habitat size **(H)**.

There was no relationship between the richness of exotic and native plant species (*r* = 0.074, *P* > 0.05) (**Figure 2A**), but the exotic plant species richness was negatively related to both the biomass (*r* = 0.358, *P* < 0.001) and the relative coverage (*r* = 0.401, *P* < 0.001) (**Figures 2B,C**) of native plants. The exotic plant species richness was positively related to the TP (*r* = 0.251, *P* < 0.001) (**Figure 2D**), TN (*r* = 0.220, *P* < 0.001) (**Figure 2E**), and COD (*r* = 0.185, *P* < 0.005) (**Figure 2F**) of the water. Neither latitude nor habitat size impacted the exotic plant species richness (**Figures 2G,H**). However, latitude was negatively related to exotic plant species richness when the analysis was limited to the communities where exotic species were recorded (*r* = 0.151, *P* < 0.05).

**FIGURE 3:**
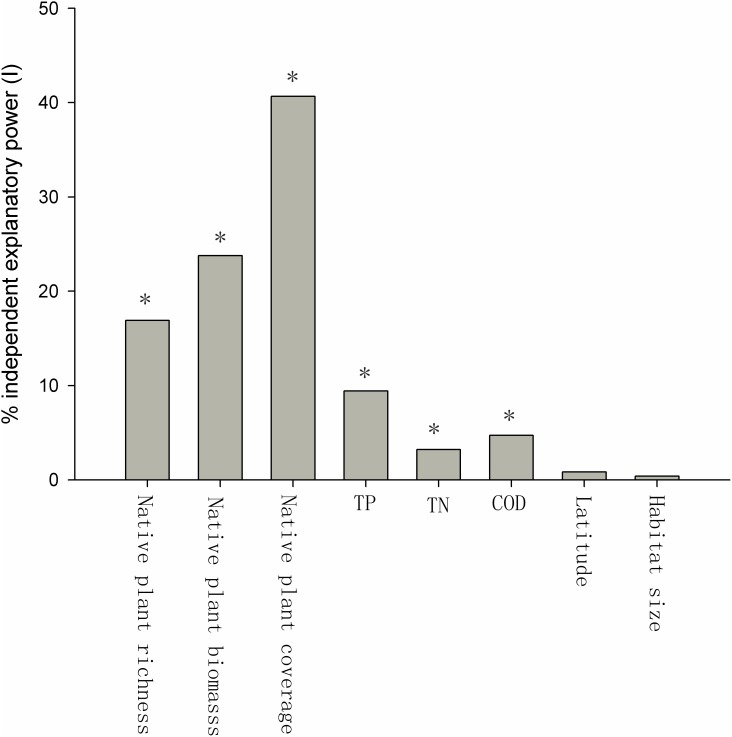
Percent independent explanatory power (*I*) of biotic and abiotic factors of exotic plant richness. The *Z*-scores were 14.21 (native plant richness), 15.42 (native plant biomass), 31.91 (native plant coverage), 3.61 (TP), 1.72 (TN), 1.92 (COD), 0.35 (latitude), and 0.14 (habitat size). ^∗^The *Z*-score is considered significant at *P* ≤ 0.05 (*Z* ≥ 1.65).

Many of the factors were correlated. The native plant species richness, the relative coverage of native plants, and the biomass of native plants were positively related to each other (**Table 1**), and the TP, TN, and COD of the water were also positively related to each other (**Table 1**). The native plant species richness, the relative coverage of native plants, and the biomass of native plants were each negatively correlated with the TP, TN, and COD (**Table 1**). Habitat size had a negative relationship with TN, but positive relationships with native plant species richness, the relative coverage of native plants, the biomass of native plants, and latitude were observed (**Table 1**). The relative coverage of native plants had the highest independent correlations with the exotic plant species richness, and its independent explanatory power for the richness of exotic plant species was 40.67%. The independent explanatory powers of the biomass and the richness of native plants species on the richness of exotic plant species were 23.77 and 16.90%, respectively. The influences of the water body’s nutrient level and COD on the richness of exotic plant species were weaker than the effects of native plants. The independent explanatory power of TP, TN, and COD on the richness of exotic plant species was 9.43, 3.23, and 4.74%, respectively. The independent explanatory power of latitude and habitat size on the richness of exotic plant species was the lowest, only 0.86 and 0.40%, respectively (**Figure 3**).

**Table 1:** Correlation coefficients (*r*-values) of simple regressions among TP, TN, COD, richness of native plants, coverage of native plants, biomass of native plants, and habitat size.

	TP	TN	COD	Richness of	Coverage of	Biomass of
				native plants	native plants	native plants
TN	0.683***					
COD	0.245***	0.336***				
Richness of native plants	-0.149*	-0.177*	-0.207***			
Coverage of native plants	-0.147*	-0.136*	-0.307***	0.410***		
Biomass of native plants	-0.206***	-0.126*	-0.218***	0.303***	0.650***	
Habitat size	-0.088	-0.125*	0.035	0.160**	0.177**	0.213***


*^∗^*P* < 0.05, ^∗∗^*P* < 0.01, ^∗∗∗^*P* < 0.001.*

## Discussion

### Impacts of Native Plants on the Richness of Exotic Plant Species

There is doubt concerning which characteristics of resident plants can resist invasions. Some researchers believe that abundant native plants can reduce the susceptibility of resident communities to invasion, but this depends on the spatial scale (Fridley et al., 2007). Local or fine scales are considered at a resolution of 10 m^2^ or less (Fridley et al., 2007), and the community sizes in our study ranged from 50 to 100,000 m^2^; therefore, the scale of our study was coarse (broad). We found that native plant species richness was neither positively nor negatively correlated with exotic plant species richness. In freshwater ecosystems, the likelihood of the occurrence of some exotic plants was positively affected by macrophyte richness, but some were negatively affected by macrophyte richness, and some plants had no relationship with native species richness (Thomaz et al., 2009; Thomaz and Michelan, 2011; Fleming et al., 2015). Capers et al. (2007) reported that invasive species richness at a fine scale is positively or negatively correlated with native species richness in some lakes but that invasive species richness in most lakes is not correlated with native species richness. Similar to our results, these authors also reported no correlation between native and exotic species richness at larger spatial scales. Therefore, there is no constant native species–exotic species richness relationship on either fine or large scales.

There are fewer exotic plant species in communities where biomass or relative coverage of native plants is high. Indeed, the vacant niche hypothesis, the biotic resistance hypothesis, and the resource fluctuation hypothesis all assert that a sufficient amount of resident plants can occupy all niches and sequester available resources and can thus competitively exclude invaders (Elton, 1958; Davis et al., 2000). Higher biomass and relative coverage of native plants use more space, nutrition, and light. Native species may resist invasion in communities only if the density or productivity is high (Capers et al., 2007; Davies et al., 2007). Even when the productivity of communities is low, more native species will accompany more exotic species (Davies et al., 2007). A previous study found that the densities and growth rates of native macrophytes play even more important roles in decreasing the invasibility of *Urochloa arrecta* than does native species richness (Michelan et al., 2013). Therefore, our results suggested that the abundance and biomass of native plant communities are more resistant to invaders than the richness of native plant species. However, biodiversity conservation is still a realistic and significant method for controlling invasive plants. Whether in an ecosystem or a plant community, higher species richness can increase the productivity and total cover of plants (Tilman et al., 1997; Waide et al., 1999; Balvanera et al., 2006). Higher plant species richness can utilize the spatial and temporal niches adequately, which results in higher functional diversity and can contribute to the higher productivity of the ecosystem (Tilman et al., 1997). In our study, both the relative coverage and the biomass of native plants were positively related to plant species richness, which suggests that plant species richness may affect invasive plants by altering ecosystem processes.

### Impacts of Eutrophication and Pollution on the Richness of Exotic Plants

The fluctuating resource hypothesis states that a plant community becomes more susceptible to invasion when the amount of unused resources increases (Davis et al., 2000). Increased nutrition will produce new niches and cause more exotic plants to invade communities. The competitive ability and fitness of exotic plants are promoted in a higher nutrient environment; therefore, increased nutrition could promote the probability of invasion success by exotic aquatic plants. The relative growth rates, photosynthetic rates, leaf nitrogen contents, and photosynthetic nitrogen-use efficiencies of aquatic exotic plants increase more intensively with increasing nutrients than do those of native species (Xie et al., 2010; Fan et al., 2013). Compared with native species, exotic aquatic plants exhibited improved reproductive rates and survival rates under conditions of high nutrient availability (Xie et al., 2010), and in warm temperate zones, eutrophication in water increased the overwintering survival rate of the aquatic invasive plant *E. crassipes* (Dong and Fan, unpublished data). In addition, enemy release favors exotics over natives in high-resource environments (Blumenthal, 2005, 2006).

Excessive increases in nutrients and sewage discharge also constitute a type of disturbance that markedly alters the species composition and often decreases the biological diversity in aquatic ecosystems (Balls et al., 1989; Hough et al., 1989; Hautier et al., 2009). Water eutrophication can lead to the rapid production of phytoplankton and other micro-organisms; dense phytoplankton populations kill the submerged plants by competing with submerged plants for ecological resources, such as nutrients, light, oxygen, and living space (Balls et al., 1989; Le et al., 2010). Our study found that increased TN, TP, and COD were all associated with reduced richness, biomass, or relative coverage of the native plant communities; therefore, water pollution and eutrophication can reduce the resistance of resident communities to invaders, likely favoring invaders directly. In addition, eutrophication is the consequence of human activities (Carpenter et al., 1998). Frequent human activities bring greater propagule pressure, leading to the invasion of resident communities by more exotic species (Vitousek et al., 1997; Lockwood et al., 2005).

### Impacts of Habitat Size on the Richness of Exotic Plants

Larger areas have more native and invasive species (Lonsdale, 1999) because of their higher spatial heterogeneity. In our study, the number of native species increased with an increase in habitat size, but there was no relationship between exotic species richness and habitat size. In our study, some plants, such as *A. philoxeroides*, *M. aquaticum*, *E. crassipes*, and *P. stratiotes*, have consistent life forms, morphologies, and niches. Higher morphological plasticity enables invasive plants to live in a variety of environments (Richards et al., 2006; Davidson et al., 2011). Therefore, some exotic aquatic plants can coexist in small habitats. We also found that habitat size had positive relationships with richness, relative coverage, and biomass of native plants but were negatively correlated with TN in the water. This result suggested that large habitats are more tolerant to disturbance than small habitats.

### Native Plant Species Are More Likely to Predict the Richness of Exotic Plant Species Than Other Factors

The identification of which factors are of greatest importance for predicting the invasion of exotic plants can help us prevent exotic species invasion and minimize their impacts (Gosper et al., 2015). Souza et al. (2011) found that native species richness rather than abiotic predictors (road density, soil moisture, and light availability) accounted for most of the variation in exotic plant richness at the landscape scale. However, at the local scale, native plant richness, light availability, road density, field density, and soil clay accounted for similar amounts of variation in exotic plant species richness. Another study also found that the independent explanatory powers of 12 predictors of the presence of alien species were similar (Gosper et al., 2015). Our study found that the independent explanatory power of native plant coverage of the exotic plant species richness was highest. Overall, biotic predictors (richness, coverage, and biomass of native plants) rather than abiotic predictors (TN, TP, and COD in water, latitude, and habitat size) accounted for more of the variation in exotic plant species richness, consistent with the results of Souza et al. (2011). Our study implied that improving the vegetation coverage and biodiversity is the most effective approach for preventing alien plant invasions and minimizing their impacts in freshwater ecosystems. The reduction of industrial and domestic wastewater discharge and hydroelectric projects, the prohibition of aquaculture, sand excavation and mining, wetland and lake corrosion, the improvement of environmental awareness, and ecological restoration will improve the vegetation coverage (Fang et al., 2006; Xu et al., 2006).

Although the coverage of native plants is the most significant factor influencing richness of exotic plant species in communities, the factors for different exotic aquatic plants were different. Increases in the water N and P concentrations were the main reason for the successful colonization of free-floating exotic plants, whereas richer nutrients in sediments enhance the chances of colonization and successful invasion of rooted macrophytes (Thomaz et al., 2015). Even though *E. crassipes*, *P. stratiotes*, and *Salvinia molesta* are all free-floating exotic plants, their response to the rise in nutrition was different (Henry-Silva et al., 2008). Warming can increase the survival percentage, regrowth, and clonal propagation of *E. crassipes* (You et al., 2013), but it did not influence the abundance or growth rate of *E. nuttallii* (Mckee et al., 2002). Aquatic vegetation loss and eutrophication in global freshwater ecosystems is becoming increasingly serious. In future research, we plan to focus on the establishment and colonization mechanisms of exotic aquatic plants in an environment of increasing nutrition and on investigating the synergistic effects of eutrophication and vegetation loss on aquatic plant invasion.

## Conclusion

Researchers pay substantial attention to studying the relationships between native and exotic species richness to assess the susceptibility of resident communities to invasion. However, our study found that the richness of native plant species cannot directly predict the number of exotic plant species in aquatic plant communities. Native aquatic vegetation loss, eutrophication, and water pollution are important factors that cause the invasion of exotic aquatic plants in freshwater ecosystems in China. China is still a developing country, and its freshwater ecosystems are under substantial strain. Strengthening the protection and restoration of fragile freshwater ecosystems is beneficial to controlling invasive species and conserving the native biodiversity.

## Author Contributions

SF and CL designed and executed the research project. HY, SF, and LW collected the field data. HY led the reflectance data analysis and drafted the manuscript with the assistance of SF. All the co-authors commented on and approved the final manuscript.

## Conflict of Interest Statement

The authors declare that the research was conducted in the absence of any commercial or financial relationships that could be construed as a potential conflict of interest.

## References

[B1] Balls H. Moss B. Irvine K. (1989). The loss of submerged plants with eutrophication I. Experimental design, water chemistry, aquatic plant and phytoplankton biomass in experiments carried out in ponds in the Norfolk Broadland. *Freshw. Biol.* 22 71–87. 10.1111/j.1365-2427.1989.tb01085.x

[B2] Balvanera P. Pfisterer A. B. Buchmann N. He J. S. Nakashizuka T. Raffaelli D. (2006). Quantifying the evidence for biodiversity effects on ecosystem functioning and services. *Ecol. Lett.* 9 1146–1156. 10.1111/j.1461-0248.2006.00963 16972878

[B3] Blumenthal D. M. (2005). Interrelated causes of plant invasion. *Science* 310 243–244. 10.1126/science.1114851 16224008

[B4] Blumenthal D. M. (2006). Interactions between resource availability and enemy release in plant invasion. *Ecol. Lett.* 9 887–895. 10.1111/j.1461-0248.2006.00934.x 16796578

[B5] Burns J. H. (2004). A comparison of invasive and non-invasive dayflowers (Commelinaceae) across experimental nutrient and water gradients. *Divers. Distrib.* 10 387–397. 10.1111/j.1366-9516.2004.00105.x

[B6] Capers R. S. Selsky R. Bugbee G. J. White J. C. (2007). Aquatic plant community invasibilty and scale-dependent patterns in native and invasive species richness. *Ecology* 88 3135–3143. 10.1890/06-1911.1 18229847

[B7] Carpenter S. R. Caraco N. F. Correll D. L. Howarth R. W. Sharpley A. N. Smith V. H. (1998). Nonpoint pollution of surface waters with phosphorus and nitrogen. *Ecol. Appl.* 8 559–568.

[B8] Clavero M. García-Berthou E. (2005). Invasive species are a leading cause of animal extinctions. *Trends Ecol. Evol.* 20:110. 10.1016/j.tree.2005.01.003 16701353

[B9] Daehler C. C. (2003). Performance comparisons of co-occurring native and alien invasive plants: implications for conservation and restoration. *Ann. Rev. Ecol. Evol. Syst.* 34 183–211. 10.1146/annurev.ecolsys.34.011802.132403

[B10] D’Antonio C. M. Kark S. (2002). Impacts and extent of biotic invasions in terrestrial ecosystems. *Trends Ecol. Evol.* 17 202–204. 10.1016/S0169-5347(02)02454-0

[B11] Davidson A. M. Jennions M. Nicotra A. B. (2011). Do invasive species show higher phenotypic plasticity than native species and, if so, is it adaptive? A meta-analysis. *Ecol. Lett.* 14 419–431. 10.1111/j.1461-0248.2011.01596.x 21314880

[B12] Davies K. F. Harrison S. Safford H. D. Viers J. H. (2007). Productivity alters the scale dependence of the diversity–invasibility relationship. *Ecology* 88 1940–1947. 17824424 10.1890/06-1907.1

[B13] Davis M. A. Grime J. P. Thompson K. (2000). Fluctuating resources in plant communities: a general theory of invasibility. *J. Ecol.* 88 528–534. 10.1046/j.1365-2745.2000.00473.x 16026814

[B14] Dukes J. S. Mooney H. A. (1999). Does global change increase the success of biological invaders? *Trends Ecol. Evol.* 14 135–139. 10.1016/S0169-5347(98)01554-7 10322518

[B15] Elton C. S. (1958). *The Ecology of Invasion by Plants and Animals.* London: Methuen.

[B16] Fan S. Liu C. Yu D. Xie D. (2013). Differences in leaf nitrogen content, photosynthesis, and resource-use efficiency between *Eichhornia crassipes* and a native plant *Monochoria vaginalis* in response to altered sediment nutrient levels. *Hydrobiologia* 711 129–137. 10.1007/s10750-013-1471-3

[B17] Fang J. Wang Z. Zhao S. Li Y. Tang Z. Yu D. (2006). Biodiversity changes in the lakes of the Central Yangtze. *Front. Ecol. Environ.* 4:369–377.

[B18] Fleming J. P. Dibble E. D. Madsen J. D. Wersal R. M. (2015). Investigation of Darwin’s naturalization hypothesis in invaded macrophyte communities. *Biol. Invasion.* 17 1519–1531. 10.1007/s10530-014-0812-0

[B19] Fridley J. D. Stachowicz J. J. Naeem S. Sax D. F. Seabloom E. W. Smith M. D. (2007). The invasion paradox: reconciling pattern and process in species invasions. *Ecology* 88 3–17. 17489447 10.1890/0012-9658(2007)88[3:tiprpa]2.0.co;2

[B20] Funk J. L. (2008). Differences in plasticity between invasive and native plants from a low resource environment. *J. Ecol.* 96 1162–1173. 10.1111/j.1365-2745.2008.01435.x

[B21] Funk J. L. Vitousek P. M. (2007). Resource-use efficiency and plant invasion in low-resource systems. *Nature* 446 1079–1081. 10.1038/nature05719 17460672

[B22] Gilbert B. Lechowicz M. J. (2005). Invasibility and abiotic gradients: the positive correlation between native and exotic plant diversity. *Ecology* 86 1848–1855. 10.1890/04-0999

[B23] Gosper C. R. Prober S. M. Yates C. J. Scott J. K. (2015). Combining asset-and species-led alien plant management priorities in the world’s most intact Mediterranean-climate landscape. *Biodivers. Conserv.* 24 2789–2807. 10.1007/s10531-015-0973-x

[B24] Hautier Y. Niklaus P. A. Hector A. (2009). Competition for light causes plant biodiversity loss after eutrophication. *Science* 324 636–638. 10.1126/science.1169640 19407202

[B25] Henry-Silva G. G. Camargo A. F. Pezzato M. M. (2008). Growth of free-floating aquatic macrophytes in different concentrations of nutrients. *Hydrobiologia* 610:153.

[B26] Hough R. A. Fornwall M. D. Negele B. J. Thompson R. L. Putt D. A. (1989). Plant community dynamics in a chain of lakes: principal factors in the decline of rooted macrophytes with eutrophication. *Hydrobiologia* 173 199–217.

[B27] Jin X. Xu Q. Huang C. (2005). Current status and future tendency of lake eutrophication in China. *Sci. Chin. Ser. C* 48 948–954. 16512216

[B28] Kennedy T. A. Naeem S. Howe K. M. Knops J. M. (2002). Biodiversity as a barrier to ecological invasion. *Nature* 417 636–638. 10.1038/nature00776 12050662

[B29] Le C. Zha Y. Li Y. Sun D. Lu H. Yin B. (2010). Eutrophication of lake waters in China: cost, causes, and control. *Environ. Manage* 45 662–668. 10.1007/s00267-010-9440-3 20177679

[B30] Levine J. M. D’Antonio C. M. (1999). Elton revisited: a review of evidence linking diversity and invasibility. *OIKOS* 87 15–26. 10.2307/3546992

[B31] Li S. J. (2006). An approach to accelerating innovative development of the lake science. *Bull. Chin. Acad. Sci.* 21 399–405. 10.1111/cts.12266 25801998 PMC4575623

[B32] Lockwood J. L. Cassey P. Blackburn T. (2005). The role of propagule pressure in explaining species invasions. *Trends Ecol. Evol.* 20 223–228. 10.1016/j.tree.2005.02.004 16701373

[B33] Lonsdale W. M. (1999). Global patterns of plant invasions and the concept of invasibility. *Ecology* 80 1522–1536. 10.2307/176544

[B34] Mac Nally R. (2002). Multiple regression and inference in ecology and conservation biology: further comments on identifying important predictor variables. *Biodivers. Conserv.* 11 1397–1401.

[B35] Matzek V. (2011). Superior performance and nutrient-use efficiency of invasive plants over non-invasive congeners in a resource-limited environment. *Biol. Invasion.* 13 3005–3014. 10.1007/s10530-011-9985-y

[B36] Mckee D. Hatton K. Eaton J. W. Atkinson D. Atherton A. Harvey I. (2002). Effects of simulated climate warming on macrophytes in freshwater microcosm communities. *Aquat. Bot.* 74 71–83.

[B37] Merow C. Boisc S. T. Allend J. M. Xiee Y. Silander J. A. Jr. (2017). Climate change both facilitates and inhibits invasive plant ranges in New England. *Proc. Natl. Acad. Sci. U.S.A.* 114 E3276–E3284. 10.1073/pnas.1609633114 28348212 PMC5402445

[B38] Michelan T. S. Thomaz S. M. Bini L. M. (2013). Native macrophyte density and richness matter for invasiveness of a tropical Poaceae. *PLoS One* 8:e60004. 10.1371/journal.pone.0060004 23536902 PMC3607602

[B39] Naeem S. Knops J. M. Tilman D. Howe K. M. Kennedy T. Gale S. (2000). Plant diversity increases resistance to invasion in the absence of covarying extrinsic factors. *OIKOS* 91 97–108. 10.1034/j.1600-0706.2000.910108.x

[B40] Richards C. L. Bossdorf O. Muth N. Z. Gurevitch J. Pigliucci M. (2006). Jack of all trades, master of some? On the role of phenotypic plasticity in plant invasions. *Ecol. Lett.* 9 981–993. 10.1111/j.1461-0248.2006.00950.x 16913942

[B41] R Development Core Team (2017). *R: A Language and Environment for Statistical Computing.* Vienna: R Foundation for Statistical Computing Available at: https://www.r-project.org/

[B42] Shao M. Tang X. Zhang Y. Li W. (2006). City clusters in China: air and surface water pollution. *Front. Ecol. Environ.* 4:353–361. 10.1890/1540-9295(2006)004[0353:CCICAA]2.0.CO;2 28895029

[B43] Shea K. Chesson P. (2002). Community ecology theory as a framework for biological invasions. *Trends Ecol. Evol.* 17 170–176. 10.1016/S0169-5347(02)02495-3

[B44] Souza L. Bunn W. A. Simberloff D. Lawton R. M. Sanders N. J. (2011). Biotic and abiotic influences on native and exotic richness relationship across spatial scales: favourable environments for native species are highly invasible. *Funct. Ecol.* 25 1106–1112. 10.1111/j.1365-2435.2011.01857.x

[B45] Stohlgren T. J. Jarnevich C. Chong G. W. Evangelista P. H. (2006). Scale and plant invasions: a theory of biotic acceptance. *Preslia* 78 405–426.

[B46] Thomaz S. M. Carvalho P. Mormul R. P. Ferreira F. A. Silveira M. J. Michelan T. S. (2009). Temporal trends and effects of diversity on occurrence of exotic macrophytes in a large reservoir. *Acta Oecol.* 35 614–620. 10.1016/j.actao.2009.05.008

[B47] Thomaz S. M. Michelan T. S. (2011). Associations between a highly invasive species and native macrophytes differ across spatial scales. *Biol. Invasion.* 13 1881–1891. 10.1007/s10530-011-0008-9

[B48] Thomaz S. M. Mormul R. P. Michelan T. S. (2015). Propagule pressure, invasibility of freshwater ecosystems by macrophytes and their ecological impacts: a review of tropical freshwater ecosystems. *Hydrobiologia* 746 39–59.

[B49] Tilman D. Knops J. Wedin D. Reich P. Ritchie M. Siemann E. (1997). The influence of functional diversity and composition on ecosystem processes. *Science* 277 1300–1302. 10.1126/science.277.5330.1300

[B50] Vilà M. Espinar J. L. Hejda M. Hulme P. E. Jarošík V. Maron J. L. (2011). Ecological impacts of invasive alien plants: a meta-analysis of their effects on species, communities and ecosystems. *Ecol. Lett.* 14 702–708. 10.1111/j.1461-0248.2011.01628.x 21592274

[B51] Vitousek P. M. (1990). Biological invasions and ecosystem processes: towards an integration of population biology and ecosystem studies. *OIKOS* 57 7–13. 10.2307/3565731

[B52] Vitousek P. M. Mooney H. A. Lubchenco J. Melillo J. M. (1997). Human domination of Earth’s ecosystems. *Science* 277 494–499. 10.1126/science.277.5325.494

[B53] Waide R. B. Willig M. R. Steiner C. F. Mittelbach G. Gough L. Dodson S. I. (1999). The relationship between productivity and species richness. *Ann. Rev. Ecol. Syst.* 30 257–300. 10.1146/annurev.ecolsys.30.1.257

[B54] Walsh C. Mac Nally R. (2013). *Package Hier. Part: Hierarchical Partitioning, Version 1.0-4.* Available at: http://cran.rproject.org/ [accessed May 6, 2014].

[B55] Xie D. Yu D. Yu L. F. Liu C. H. (2010). Asexual propagations of introduced exotic macrophytes *Elodea nuttallii*, *Myriophyllum aquaticum*, and M. *propinquum* are improved by nutrient-rich sediments in China. *Hydrobiologia* 655 37–47. 10.1007/s10750-010-0402-9

[B56] Xu Q. J. Jin X. C. Yan C. Z. (2006). Macrophyte degradation status and countermeasures in China. *Ecol. Environ.* 15 1126–1130. 10.16258/j.cnki.1674-5906.2006.05.047

[B57] Yang X. Pang J. (2006). Implementing China’s “Water Agenda 21”. *Front. Ecol. Environ* 4 362–368.

[B58] You W. Yu D. Xie D. Yu L. (2013). Overwintering survival and regrowth of the invasive plant *Eichhornia crassipes* are enhanced by experimental warming in winter. *Aquat. Biol.* 19 45–53. 10.3354/ab00519

